# High-Level Recombinant Human Lysozyme Expressed in Milk of Transgenic Pigs Can Inhibit the Growth of *Escherichia coli* in the Duodenum and Influence Intestinal Morphology of Sucking Pigs

**DOI:** 10.1371/journal.pone.0089130

**Published:** 2014-02-21

**Authors:** Dan Lu, Qiuyan Li, Zhibin Wu, Shengzhe Shang, Shen Liu, Xiao Wen, Zhiyuan Li, Fangfang Wu, Ning Li

**Affiliations:** 1 The State Key Laboratory for Agro-biotechnology, China Agricultural University, Beijing, China; 2 Beijing Genfucare Biotechnology Company, Beijing, China; 3 College of Animal Science and Technology, Yunnan Agricultural University, Kunming, China; University of Connecticut, United States of America

## Abstract

Lysozyme is often used as a feed additive and acts as an antimicrobial protein that enhances immune function and defends against pathogenic bacteria in pigs. In this study, we genetically added recombinant human lysozyme (rhLZ) to sow milk by somatic cell nuclear transfer and investigated whether the presence of recombinant human lysozyme can influence intestinal microbiota and mophology in sucking pigs. We generated transgenic cloned pigs and the first-generation hybrids (F1) produced high levels of rhLZ in milk. The average concentration of rhLZ was 116.34±24.46 mg/L in the milk of F1 sows, which was 1500-fold higher than that of the native pig lysozyme. In vitro, it was demonstrated that rhLZ in milk of transgenic pigs had enzyme levels at 92,272±26,413 U/mL. In a feeding experiment, a total of 40 newborn piglets were nursed by four transgenic sows and four sibling non-transgenic sows (F1), with five piglets per gilt. The piglets were allowed to nurse for 21 days and the sow milk was the only source of nutrition for the piglets. All piglets were slaughtered on postnatal day 22. Six types of bacteria were cultured and analyzed to detect the impact of rhLZ on gut microbiota. The number of *Escherichia coli* in the duodenum of piglets reared by transgenic sows was significantly decreased (*p*<0.001) and their villus height to crypt depth ratio in the intestine were increased due to the significant decrease of crypt depth in the duodenum, jejunum, and ileum (*p*<0.001). Together, we successfully generated rhLZ transgenic cloned pigs and elevated lysozyme level in nuring piglets. The results of the feeding experiments demonstrated that rhLZ-enhanced milk can inhibit the growth of *E. coli* in the duodenum and positively influence intestinal morphology without adversely affecting weight gain or piglet growth.

## Introduction

One of the greatest aspirations in modern biology is the ability to utilize the ever expanding knowledge of the genetic basis of the enormous phenotypic diversity that exists in contemporary livestock and other organisms. Owing to the development of transgenic technologies, researchers are now able to produce transgenic animals, including livestock species, as specific biomedical research models for various human afflictions, including Alzheimer’s disease, cystic fibrosis, diabetes, and providing tissues for xenotransplantation [1]–[4]. Transgenic animals are also currently used to study animal diseases, such as prion diseases that causing scrapie in small ruminants and bovine spongiform encephalopathy (BSE) [5], [6] and udder infections (mastitis) in dairy cows and goats [7], [8]. Animal breeding to achieve disease resistance by gene modification of complement is the traditional tactic to combat disease and provide novel interventive strategies, since it is orientable and can shorten breeding time. The work reported here is an initial step in breeding pigs for diarrhea resistance by specifically expressing human lysozyme in mammary gland tissue to benefit piglet health and improve their ability to resist bacterial infections.

Both suckling and weaning piglets are more susceptible to diarrheal diseases. Diseased pigs generally appear anorexic, gaunt, and their feces are light brown, loose to watery, and contain mucus and/or undigested feed [9]. The influence of diarrhea on surviving piglets manifests as growth retardation, lighter weaning weight, reduced feed efficiency, and lower slaughtering rate, which collectively lead to a significant negative impact on the development and economic benefit to the swine industry. However, the use of conventional antibiotics to combat pathogens is restricted, since their use could promote the development of antibiotic-resistant strains and antibiotic residues may be harmful to human health [10], [11]. Therefore, an increasing amount of research has focused on the use of natural antibacterial proteins, such as lysozyme, as an antibiotic-free approach to treat bacterial infections. Human lysozyme transgenic rice or purified lysozyme have also shown the same effects as conventional antibiotics to promote growth in poultry [12] or swine [13], [14]. Lysozyme is an important, non-specific, antimicrobial protein that widely exists in microorganisms, plants, and animals, and shows strong antimicrobial activity against Gram-positive bacterial species by hydrolyzing the glycosidic β-(1–4) linkage between N-acetylmuramic acid and N-acetylglucosamine of the peptidoglycan polymer in the bacterial cell wall [15]. Besides antimicrobial ability, lysozyme also displays anti-inflammatory capabilities by decreasing the expression of pro-inflammatory cytokines and increasing the expression of anti-inflammatory cytokines [16], and immune regulatory function by directly and indirectly modulating the complement system [17].

The concentration of lysozyme in human milk is about 400 mg/L [18], which is 1500–3000-fold greater than that found in the milk of other mammals, such as cattle and goats. However, the lysozyme concentration in pigs is even lower, less than 0.065 mg/L [19]. In comparison to other species, human lysozyme possesses the highest enzymatic activity, which is 2–3-fold greater than that of hen egg lysozyme [20]. Recent studies have indicated that consumption of milk from transgenic goats that contained recombinant human lysozyme (rhLZ) can potentially benefit microbe enrichment and reduce detrimental microbe content in the gut flora [21]. Consumption of goat rhLZ milk can also accelerate the recovery of piglets from bacterial-induced diarrhea [22] and improve their gastrointestinal health [23]. Studies on the response of weaned piglets to an oral challenge of *Escherichia coli* strain K88 indicated that piglets showed better intestinal growth and development, as well as decreased enterotoxigenic *E. coli* counts in the intestinal mucosa and serum proinflammatory cytokines following lysozyme supplementation [24].

Based on these previous studies, we reasoned that rhLZ expressed in the mammary glands of transgenic pigs could benefit the health of sucking piglets and also improve growth of weaning piglets. Our previous study demonstrated that rhLZ transgenic pigs, generated using the plasmid pBC1-hLZ-GFP-Neo, showed lower levels of rhLZ expression in pig milk relative to that in human milk [25], which has less breeding value. However, the plasmid pBC2-HLY-NEOR successfully expressed bioactive rhLZ in cow milk with a concentration of 25.96 mg/L and rhLZ showed the same physicochemical properties, such as molecular mass and bacterial lysis, as its natural counterpart [20]. Therefore, we generated transgenic pigs expressing plasmid pBC2-HLY-NEOR to increase the lysozyme expression in pig milk and then assessed whether rhLZ-enhanced milk influenced the intestinal microbiota and morphology of sucking pigs and improved protection to piglets from birth to weaning.

## Materials and Methods

### Ethics Statement

All animal procedures were approved by the Institutional Animal Care and Use Committee of the China Agricultural University (Beijing, China). All surgeries were performed under sodium pentobarbital anesthesia to minimize suffering of the animals.

### Generation of Transgenic Pigs

**Figure 1:**
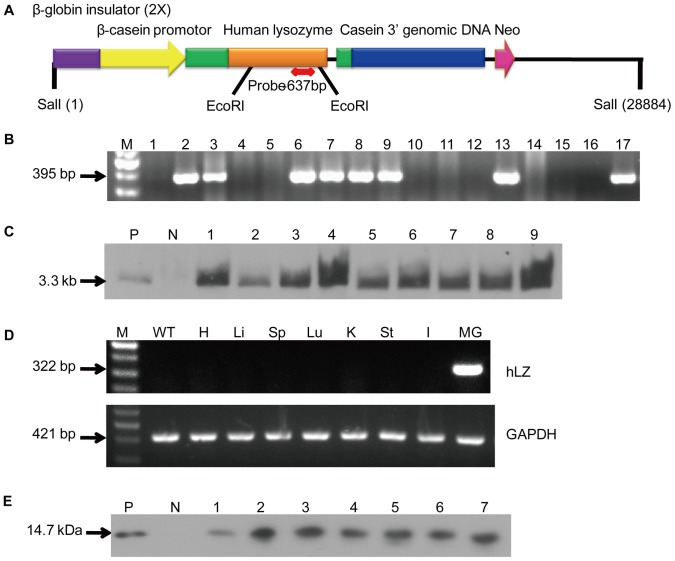
Generation and molecular characterization of transgenic pigs. (A) A schematic of pBC2-HLY-NEOR. The lower bars indicate the lengths of the PCR products and the primer pairs used for DIG-labeling of the probes to screen transgenic pigs. Globin insulator (2X), two copies of chicken β-globin insulator; β-casein promoter, goat β-casein promoter; human lysozyme, hLZ coding region; casein 3′ genomic DNA, goat β-casein 3′ genomic region. (B) PCR analysis of transgenic pigs. M, 1-kb DNA ladder; lanes 1–13, genomic DNA from transgenic pigs in the following order: 0201, 0203, 0205, 0207, 0301, 0303, 0305, 0307, 0309, 0311, 0313, 0315, and 0317; lanes 14–16, genomic DNA from three non-transgenic pigs; P, a positive control vector. (C) Southern blot identification of transgenic pigs. The bands were hybridized to a 637-bp specific probe that is complementary to a fragment of the hLZ gene. P, plasmid vectors as the positive control; N, genomic DNA from a non-transgenic pig; lanes 1–2, genomic DNA from F0 transgenic pigs 0303 and 0309; lanes 3–9, genomic DNA from F1 transgenic pigs in the following order: 0302, 0402, 0906, 1708, 1916, 0504, and 1504. (D) RT-PCR analysis of transgenic pigs. The 322-bp fragments represent the RT-PCR products of rhLZ and the 421-bp fragments represent the RT-PCR products of GAPDH. M, 1-kb DNA ladder; WT, wild-type pig mammary gland; H, transgenic pig heart; Li, liver; Sp, spleen; Lu, lung; K, kidney; St, stomach; I, intestine; and MG, mammary gland. (E) Western blot analysis of transgenic pigs. The milk samples collected on lactation day 1 were separated by SDS-PAGE and hybridized with anti-hLZ. P, commercial hLZ as a positive control (200 ng); N, non-transgenic milk; lanes 1–7, diluted milk from transgenic pigs (1∶3, 3 µL) in the following order: 0302, 0402, 0404, 0906, 1708, 1914, and 1916.

The rhLZ expression vector pBC2-HLY-NEOR (Figure 1A), which was expressed previously in transgenic cattle [20], was used to generate transgenic pigs. *Sal*I-digested fragments containing the hLZ sequence from the pBC2-HLY-NEOR expression vector were transfected into fetal fibroblasts obtained from Large White piglets, and positive clones were selected using G418 (geneticin). Nuclear transfer was performed as described previously [26], [27]. Briefly, the nuclei of transgenic cells were transferred to enucleated oocytes to produce reconstructed embryos, which were then fused and activated simultaneously by application of two direct current pulses of 1.6 kV/cm for 100 µs each at an interval of 1 s using a BTX 2001 Electro Cell Manipulator (BTX, Inc., San Diego, CA, USA) in activation medium (0.3 M mannitol supplemented with 0.05 mM CaCl_2_, 0.1 mM MgCl_2_, and 0.01% polyvinyl alcohol in H_2_O). After chemical activation with 2.5 µg/mL cytochalasin B and 10 µg/mL cycloheximide in porcine zygote medium-3, day-2 blastocysts were transferred to synchronous recipient sows with hundreds of embryos per recipient.

### Polymerase Chain Reaction (PCR) and Southern Blot Analyses

Chromosomal DNA was isolated from the tails of offspring and transgenic pigs were detected by PCR. The primers sequences are presented in Table S1 in File S1. Briefly, 10 mg of genomic DNA digested by *Eco*RI was used to detect transgenic pigs by Southern blot analysis. Non-transgenic porcine genomic DNA was used as a negative control and the pBC2-HLY-NEOR plasmid was used as a positive control. A digoxigenin (DIG)-labeled probe (Roche Diagnostics GmbH, Mannheim, Germany) was generated by PCR using the P-HLZ-637 primer and the positive hybridization signal was a 3.3-kb fragment.

### Reverse Transcription (RT)-PCR

Total RNA was extracted from multiple tissues using Trizol reagent (Tiangen, Beijing, China). First strand complementary DNA was synthesized using oligo-dT (Promega Corporation, Madison, WI, USA). RT-PCR primers were designed on the basis of the hLZ coding sequences and the upstream primer P-HLZ-322 was designed across one intron. The predicted 322-bp fragment was amplified. Pig glyceraldehyde-3-phosphate dehydrogenase (GAPDH) was used as an internal control. The primers for pig GAPDH amplified a 421-bp fragment.

### Quantitative Real-time PCR (qPCR)

qPCR was used to detect copy number as described previously [28] and all reactions were performed in 96-well plates using the Roche LightCycler 480 System (Roche, Basel, Switzerland). The amplification was performed in a 20-µL reaction volume containing 1 µL of template DNA (10 ng/µL), 0.3 µL of each primer (10 µM), 10 µL of Power SYBR Green Mix (Applied Biosystems, Inc., Foster City, CA, USA), and 8.4 µL of ddH_2_O. All reactions were performed using the following cycle conditions: 95°C for 10 min; 40 cycles at 95°C for 10 s, 60°C for 10 s, and 72°C for 10 s; followed by 95°C for 5 s, 65°C for 1 min, and then 97°C continuously to generate a melting curve. The standard curve was established using a set of standards representing 1, 2, 4, 8,16, and 32 plasmid DNA copies in 10 ng of wild-type (WT) pig genomic DNA. The *myostatin* gene (MSTN; gene ID, 399534), which has one copy in the pig genome, served as an internal control to calculate transgene copy number.

### Milk Sample Collection

Milk samples from transgenic pigs were collected within 6 h of the completion of farrowing (0 h) and 12, 24, and 48 h postpartum to analyze rhLZ expression in colostrum. Milk samples also were collected on lactation days 3, 7, 14, and 21 to analyze rhLZ expression in mature milk. All milk samples were aliquoted and stored at −20°C until assayed.

### Western Blot Analysis

Milk samples from transgenic pigs collected on lactation day 1 were diluted three-fold with distilled water and defatted by centrifugation (10,000×*g*, 15 min, 4°C). The skim milk was resolved by 15% sodium dodecyl sulfate polyacrylamide gel electrophoresis and then electrophoretically transferred to a nitrocellulose membrane (Amersham Pharmacia UK, Ltd., Buckinghamshire, UK) and blocked overnight at 4°C with 3% bovine serum albumin in phosphate-buffered saline containing 0.05% (w/v) Tween 20. Polyclonal rabbit anti-hLZ (1∶2000) (US Biological Inc., Swampscott, MA, USA) and horseradish peroxidase-conjugated goat anti-rabbit IgG (1∶20000) (Sino-American Co., Beijing, China) were used to detect rhLZ. Milk samples from WT pigs served as negative controls. hLZ standards (Sigma-Aldrich, St. Louis, MO, USA) served as positive controls. Blots were developed by enhanced chemiluminescence and autoradiography.

### rhLZ Quantification using an Enzyme-linked Immunosorbent Assay (ELISA)

The amount of rhZ in the milk of transgenic pigs collected at different time points was quantified using a Human Lysozyme ELISA kit (Biomedical Technologies, Inc., Stoughton, MA, USA). Data were collected from three independent experiments and the results are presented as means ± standard deviations.

### Lysozyme Activity Assays

*Micrococcus lysodeikticus* cells (China General Microbiological Culture Collection Center, Beijing, China) were revived and prepared for the gel diffusion and turbidimetric assays, which were used to evaluate rHLZ activity in the milk of transgenic pigs. A suspension of *M. lysodeikticus* with mixed solid culture medium containing 1.5% nutrient broth agar (Sigma-Aldrich) was used as the medium for the gel diffusion assay. Quantitative filter paper discs (6-mm diameter) were placed on the agar plates, which were loaded with milk samples and then incubated at 28°C for 24 h. The results were assessed by measuring the inhibition zones around the filter paper discs.

The turbidimetric assay, as described by Shugar [29], was used to monitor the reduction in turbidity of a suspension of *M. lysodeikticus* cells at an absorbance of 450 nm (A450). Briefly, 2.5 mL of *M. lysodeikticus* cell suspension (A_450_, 0.60–0.7) as the substrate was prepared at 25°C in 66 mM potassium phosphate buffer (pH 6.24) and then placed in a 4-mL cuvette at room temperature. The reaction was initiated by adding 100 µL of 1∶100 dilutions of pig milk samples (test group) or 100 µL of ddH_2_O (blank group). A_450_ was recorded at 30-s intervals over a 5-min period. The ΔA_450_ per minute was used as the maximum linear rate for all groups. One unit will produce a ΔA_450_ of 0.001 nm/min at pH 6.24 at 25°C in a 2.6-mL reaction mixture. All samples were measured in triplicate.

### Composition Analysis of Milk

Up to 50 mL of colostrum (3–6 h) and mature milk (14 days) were collected from six transgenic pigs and three non-transgenic pigs for analysis of gross milk composition, including fat, protein, and lactose. All testing was completed by the Beijing Research Institute for Nutritional Resources, which has established the criteria and methods used for analysis of large quantities of biochemical and nutritional components. Colostrum and milk composition was measured according to the national food safety standard of raw milk issued by the Chinese Ministry of Health (GB19301-2010).

### Animals and Feeding Experiment

As female offspring of the founder animals attained puberty, transgenic and sibling non-transgenic gilts (F1) were bred to non-transgenic Large White boars. Of the gilts bred, four transgenic and four non-transgenic (control) gilts that conceived and farrowed successfully were used in this study. On day 107 of pregnancy, the gilts were moved into a farrowing house and monitored for signs of parturition. At parturition, gilts and their newborns were monitored during farrowing to ensure safe delivery and successful nursing afterward. Within 24 h of farrowing, litters were standardized to five piglets with similar body weights per sow and were allowed to nurse for 21 days. During the 21-day lactation period, the sow’s milk was the only source of nutrition for the piglets. All 40 piglets had free access to water and were weighed every 2 days from the third day after farrowing.

### Necropsy and Sample Collection

All piglets were slaughtered for necropsy at 22 days of age. Blood samples were obtained by anterior vena cava puncture and captured in 6-mL anticoagulant-free Vacutainer tubes (Becton Dickinson and Company, Franklin Lakes, NJ, USA) and then centrifuged at 3000×g for 10 min to obtain serum. The serum samples were stored at −80°C. The contents of the duodenum, jejunum, and ileum were collected separately for microbial analysis. The mid sections of the duodenum, jejunum, and ileum tissues were collected, flushed with a 0.9% physiological saline, immersed in 4% paraformaldehyde, and stored at 4°C for future microscopic assessment of mucosal morphology.

### Microbial Analysis

In vitro survival of *Salmonella*, *E. coli*, *Bifidobacterium*, *Lactobacillus*, total aerobes, and total anaerobes was assessed using methods previously described [29]. Briefly, 1.0 g of digesta was taken from each sample and serially diluted with 9 mL of sterile physiological saline, resulting in dilutions ranging from 10^−1^ to 10^−6^ for enumeration. Then, 0.1 mL of two or three consecutive dilutions of each sample were plated on appropriate medium. The morphology of the flora and individual cells, Gram staining, and oxygen consumption were used to identify the bacteria. Two replicates and one blank control were produced from each sample plate. The number of microbes was expressed as log_10_ colony-forming units (CFU)/g.

### Histology

Intestinal samples were fixed in 4% paraformaldehyde, embedded in paraffin, and sliced to 4-µm thick sections, which were stained with hematoxylin and eosin. Villus height, crypt depth, and the villus height to crypt depth ratio were measured at 40× magnification using Image-Pro Plus software (Image-Pro Plus 6.0; Media Cybernetics, Silver Spring, MD, USA). Ten, well-oriented, intact villi were selected and measured in triplicate for each pig.

### Statistical Analysis

All experimental data were analyzed using SPSS software (ver. 19.0; SPSS Inc., Chicago, IL, USA). A probability (*p*) value <0.05 was considered statistically significant.

## Results

### Generation of Cloned Transgenic Pigs and Transgene Transmission

The transgene vector, pBC2-HLY-NEOR, contained a 5,238-bp genomic DNA fragment of hLZ, a bovine β-casein peptide DNA sequence signal, and the neomycin resistance gene (Neor) as a selection marker (Figure 1A), which was previously used successfully to generate transgenic cattle. After somatic cell nuclear transfer, 380 embryos were transferred into two recipient gilts (one with 200 and the other with 180). Thirteen male pigs were born. By PCR analysis, hLZ was integrated into the genome of seven transgenic pigs (Figure 1B). The intact transgenic constructs in all cloned pigs was also examined by PCR (data not shown). Five piglets, including one transgenic pig, died within a few hours after birth, and eight piglets survived and were healthy after weaning. Since the founder transgenic pigs were male, the first filial (F1) generation was raised to acquire milk samples after the F1 transgenic sows farrowed. Two transgenic boars with intact transgenic constructs (0303 and 0309) from the same litter were used to generate the F1 generation piglets by mating with 17 non-transgenic Large White sows. Then, 11 F1 transgenic sows and four non-transgenic gilts, as controls, were breed with non-transgenic boars to generate the F2 offspring. Two founder boars (0303 and 0309) and samples from the bred F1 pigs were subjected to Southern blot analysis (Figure 1C). qPCR analysis showed that the average copy number of inserted hLZ gene sequences in the genome of the F0 and F1 generations was 1.98±0.21. The proportions of rhLZ-positive transgenic offspring of the F1 and F2 generations were 46.71% (71/152) and 48.75% (39/80), respectively. As expected, the transgene segregated equally among male and female offspring (Table 1).

**Table 1:** Transgene transmission and segregation in human lysozyme transgenic pigs.

Year	2009	2011	2012
Generation	F0	F1	F2
Viable offspring	13	152	80
Females born (%)^1^		79 (52)	45 (56)
Males born (%)	13 (100)	73 (48)	35 (44)
Transgenic offspring (%)	7 (54)	71 (47)	39 (49)
Transgenic females (%)		36 (50)	21 (54)
Transgenic males (%)	7 (54)	35 (50)	18 (46)

1 Percentage of transgenic animals.

### rhLZ Expression

Transgene expression in transgenic female pigs was assessed by RT-PCR using RNA isolated from lactating mammary gland tissue and eight other tissues (heart, liver, spleen, lung, kidney, stomach, intestine, and muscle). Total RNA was also isolated from the mammary gland tissue of a non-transgenic pig as a negative control. The pig *GAPDH* gene was used as a control for mRNA extraction and loading. As expected, hLZ mRNA was only detected in the mammary gland tissues of transgenic pigs during the middle of the lactation period, but not in other tissues or WT pig mammary gland tissue (Figure 1D). hLZ proteins in milk samples collected from the F1 transgenic pigs at 24 h after farrowing were detected by western blot analysis. Milk from all F1 transgenic pigs contained an expected band of about 14.7 kDa, which is the molecular weight of the natural hLZ standard (Figure 1E). rhLZ was not detected in the milk samples of the non-transgenic gilts.

### rhLZ Concentration and Activity

**Figure 2:**
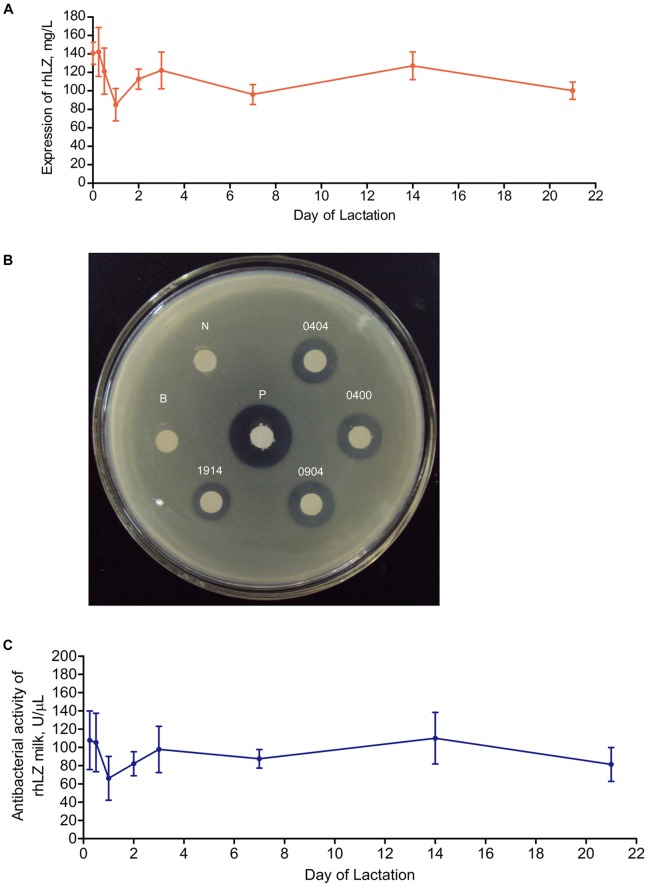
rhLZ expression level and enzymatic activity in the milk of transgenic pigs. (A) Human lysozyme concentrations in mammary secretions of first-parity transgenic gilts. Values are presented as means ± standard deviations. n = 5 gilts at each time point. (B) The gel diffusion assay for lytic activity of rHLZ milk against *Micrococcus lysodeikticus*. The small white circles are 6-mm quantitative filter papers spotted with 6 µL of 1∶3 diluted milk samples. Transparent zones around the quantitative filter papers indicate bacterial lysis. P, commercial hLZ (1 µg); B, sterile water; N, milk from non-transgenic pig; Milk samples nos. 0404, 0400, 0904, and 1914 are from F1 transgenic pigs. (C) The turbidimetric assay for antibacterial activity of rhLZ milk during lactation. Values are presented as means ± standard deviations. n = 5 gilts at each time point.

rhLZ was present in the milk samples from transgenic gilts and showed stable expression levels on all assessed days of lactation (Figure 2A). The average rhLZ concentration from five transgenic pigs during lactation was 116.34±24.46 µg/mL. The highest average concentration was 142.07±26.42 µg/mL present at 6 h postpartum (Table S2 in File S1). On lactation days 3–21, the average rhLZ concentrations did not decline much compared to the average colostrum concentration, which fluctuated around 110 µg/mL.

rhLZ activity of all transgenic pigs was examined using the gel diffusion assay, with water and milk from non-transgenic pig as negative controls and 1.0 µg of natural hLZ standard (Sigma-Aldrich) as a positive control. Transparent zones around filters containing the natural hLZ standard or milk from transgenic pigs were clearly visible from the culture medium after incubation for 24 h (Figure 2B). No transparent zone was formed by milk samples from the non-transgenic pig. By measuring the diameter of the clear zone, rhLZ activity from transgenic pigs was much lower than the activity of 1 µg of the natural hLZ standard, which was consistent with the western blotting and ELISA results.

To quantify rhLZ activity, milk samples collected from five transgenic pigs and four non-transgenic pigs were examined using the turbidimetric assay. The average rhLZ activity during the lactation period was 92,272±26,413 U/mL. The highest average activity was 110,076±28,238 U/mL on day 14 (Figure 2C and Table S2 in File S1). The general trend of average rhLZ concentration and activity during the lactation period was quite consistent. Since the turbidimetric assay can not distinguish between endogenous pig lysozyme and rhLZ, we detect the enzymatic activity of milk with three times dilution collected from four non-transgenic lactating sows. The non-transgenic milk collected at 6, 12 and 24 h showed little enzymatic activity, the average value were 84.65±6.66 U/mL, 69.59±11.40 U/mL and 3.65±0.42 U/mL, respectively. But we can not detect any enzymatic activity of milk collected after 48 h (Table S3 in File S1).

### Raw Milk Component in Samples from First-parity Controls and Transgenic Gilts

Analysis of fat, protein, and lactose content as well as concentrations of 16 different amino acids showed no significant differences in milk samples from first-parity non-transgenic controls and transgenic gilts (Tables 2 and S4 in File S1).

**Table 2:** Raw components of transgenic milk compared to conventional milk.

Item	Period	Transgenic(g/100 g)	Non-Transgenic(g/100 g)
Protein	Colostrum	17.133±2.64	15.97±2.28
	Milk	5.05±0.33	5.04±0.25
Fat	Colostrum	4.17±1.02	5.19±0.81
	Milk	6.66±1.04	7.27±1.34
Lactose	Colostrum	2.45±0.16	2.52±0.08
	Milk	4.36±1.24	4.86±0.13

No significant differences were detected between the transgenic and non-transgenic groups (*p*>0.05). Data are presented as averages ± standard deviations. Transgenic, n = 6; non-transgenic, n = 3.

### Weight and Growth of Piglets

To determine whether the presence of the human lysozyme transgene in first-lactation gilts influenced litter growth and health, we divided 40 piglets into two feeding groups nursing from four transgenic and four non-transgenic gilts separately and weighted them every 2 days from the third day after farrowing. There was no significant difference between the average mass of piglets nursed by transgenic and non-transgenic sows on days 3 and 21 (Table 3).

**Table 3:** Body weight of two piglet groups on neonatal days 3 and 21.

Item	Non-transgenic	Transgenic
Mass on day 3 days (kg)	1.56±0.38	1.58±0.29
Mass on day 21 days (kg)	5.87±0.68	5.82±0.74
Average daily weight gain (kg) gain	0.23±0.03	0.24±0.03

No significant differences were detected between the transgenic and non-transgenic groups (*p*>0.05). Values are presented as averages ± standard deviations. n = 20 for each group.

### The rhLZ Milk Reduced the Number of *E. coli* in the Duodenum of Piglets in the Feeding Experiment

All piglets were slaughtered at 22 days of age and their intestinal contents were collected for microbial analysis using a culture-based bacterial assessment method. Six types of bacteria were selected for culturing: total aerobes, total anaerobes, *Salmonella spp., E. coli, Bifidobacterium spp.*, and *Lactobacillus spp.* The results showed that number of *E. coli* in the duodenum of the experimental group was significantly decreased (*p*<0.001), but there was no difference in bacterial content in the jejunum, ileum, and colon. We observed no differences in the content of the remaining five types of bacteria among different intestinal segments of piglets in the two feeding groups (Table 4).

**Table 4:** Six types of bacteria were counted (logCFU/g of intestinal contents) in the duodenum, jejunum, ileum, and colon of piglets.

Item	Non-transgenic	Transgenic	*p*-value
Total Anaerobes			
Duodenum	11.45±0.49	10.78±0.52	0.110
Jejunum	10.28±0.57	9.96±0.48	0.421
Ileum	10.01±0.25	11.33±0.14	0.071
Colon	10.74±0.29	10.48±0.16	0.171
Total Aerobes			
Duodenum	9.93±0.39	9.83±0.34	0.704
Jejunum	9.29±0.89	9.05±0.10	0.622
Ileum	8.56±0.31	8.68±0.41	0.633
Colon	10.39±0.34	10.23±0.18	0.437
*Salmonella*			
Duodenum	6.45±0.55	6.14±0.47	0.423
Jejunum	6.21±0.42	6.11±0.71	0.809
Ileum	6.74±0.25	6.35±0.71	0.357
Colon	6.12±0.59	5.84±0.70	0.566
*Escherichia coli*			
Duodenum	7.62±0.24	6.56±0.17	<0.001
Jejunum	7.00±0.39	6.85±0.66	0.707
Ileum	7.70±0.39	7.58±0.43	0.691
Colon	7.09±0.35	6.77±0.34	0.236
*Bifidobacterium*			
Duodenum	6.92±0.56	6.69±0.16	0.477
Jejunum	7.11±0.76	6.77±0.42	0.465
Ileum	7.04±0.68	6.80±0.67	0.645
Colon	7.29±0.34	6.98±0.33	0.239
*Lactobacillus*			
Duodenum	8.16±0.19	8.22±0.27	0.722
Jejunum	8.55±0.61	8.92±0.10	0.271
Ileum	8.43±0.28	8.70±0.37	0.280
Colon	9.34±0.25	8.99±0.50	0.265

Values are presented as averages ± standard deviations.

### Lysozyme Transgenic Milk Influenced Gastrointestinal Morphology in Young Pigs

To evaluate the influence of lysozyme transgenic milk on gastrointestinal morphology in young pigs, villus height, crypt depth, and the villus height to crypt depth ratio were measured (Table 5). We observed no significant difference in villus height among all intestinal segments between piglets nursed by transgenic sows and those by non-transgenic sows. However, the crypt depths of piglets nursed by non-transgenic sows were deeper than those nursed by transgenic sows in the duodenum (*p*<0.001), jejunum (*p*<0.001), and ileum (*p*<0.001). In the jejunum, the villus height and the villus height to crypt depth ratio of piglets nursed by transgenic sows were much higher than those from the control group, although there were no significant differences (*p* = 0.055 and 0.074, respectively).

**Table 5:** Histological measurements from the duodenum, jejunum, ileum of piglets.

Item	Non-transgenic	Transgenic	*p*-value
Duodenum			
Villus height, µm	445.54±12.19	453.80±8.64	0.581
Crypt depth, µm	192.69±3.28	176.68±3.09	<0.001
Villus height:crypt depthratio	2.33±0.19	2.58±0.14	0.301
Jejunum			
Villus height, µm	437.02±13.96	471.19±10.81	0.055
Crypt depth, µm	158.23±2.63	139.01±3.33	<0.001
Villus height:crypt depthratio	2.78±0.29	3.43±0.18	0.074
Ileum			
Villus height, µm	386.52±11.73	357.25±11.12	0.072
Crypt depth, µm	141.61±3.43	118.21±2.10	<0.001
Villus height:crypt depthratio	2.92±0.40	3.10±0.34	0.733

Values are presented as averages ± standard error of the mean.

## Discussion

We have sucessfully produced transgenic pigs with human lysozyme expressing in milk at 1500-fold higher than that of the native pig lysozyme and greatly elevated lysozyme level in nursing piglets. This report is the first to date regarding the influence of in vivo rhLZ on piglets nursed by rhLZ transgenic sows under naturalistic conditions.

A previous study demonstrated that high rhLZ concentrations (1.405 g/L) can be expressed in mouse mammary gland tissues [30], but the rhLZ concentrations in pig and bovine mammary gland tissues were quite lower than expected [20], [25]. However, in the present study, we successfully generated transgenic pigs with high rhLZ expression levels, which was much higher than that achieved in previous studies. We also showed that the expression plasmid pBC2-HLY-NEOR efficiently expressed rhLZ in pigs. In contrast to the results of previous reports, these differences may due to the integrity of gene control regions and the position effect. We have already obtained the F2 generation, and the proportion of rhLZ-positive transgenic pigs in each generation approached a ratio of 1∶1 (F1 = 46.71% and F2 = 46.91%). Segregation analysis indicated a typical Mendelian inheritance, suggesting a single locus or closely linked loci within the gene insertion.

rhLZ expression was stable and persistent during the lactation period of transgenic pigs. The hLZ gene and its expression were heritable from the founder transgenic animals to their offspring. The rhLZ transgenic pig line used in the present study was hemizygous and produced over 1500 times more lysozyme than what is normally found in pig milk, but there was still a gap between rhLZ expression in human milk and the hLZ transgenic pig milk. We have already obtained homozygous F2 generation piglets and anticipate that rhLZ expression could be even higher.

Expression of heterologous proteins may influence milk composition and even impair mammary gland development. Reportedly, transgenic goats expressing recombinant human butyrylcholinesterase led to a reduction in casein and short chain fatty acids, an increase in saturated fatty acids, and also altered the concentrations of Na^+^ and K^+^ [31]. Transgenic mice expressing recombinant human protein C showed physiological problems in lactation and failed to lactate normally and nurse their litters [32]. However, there were no significant or apparent changes in milk containing rhLZ produced by transgenic cows and goats [20], [33], [34]. Since changes in milk composition are strictly dependent on the introduced genetic modification, it becomes apparent that the impact on milk composition and lactation performance must be considered on a case-by-case basis. In our study, the gross composition colostrum and milk and concentrations of 16 amino acids showed no significant differences between transgenic and non-transgenic pigs, which indicated that rhLZ did not influence milk composition.

During the feeding experiment, there were no significant differences in overall weight gain and mean daily growth between the experimental and control groups. These results were similar to the findings of a study on piglets fed rhLZ transgenic goat milk [23] and demonstrated that high rhLZ concentrations present in pig milk had no adverse impact on piglet growth. It also illustrated that lactation performance was not affected since piglet growth was directly associated with milk production. Therefore, there was no detrimental influence on the lactation performance of transgenic sows and growth of piglets during the feeding experiment due to the presence of rhLZ in the milk.

Intestinal microbiota plays an important role in maintaining intestinal health and influences the normal structural and functional development of the mucosal immune system [35], [36]. Milk is the only nutrition source for newborn mammals and undoubtedly plays a crucial role in shaping the gut flora. Lysozyme as a natural antibacterial protein do some contribution to the immunological characteristics of milk, that favoring the growth of beneficial bacteria such as *Bifidobacteria* [37] and defending newborns against pathogenic infections. Pigs consuming pasteurized milk from hLZ transgenic goats show fewer numbers of coliform bacteria and *E. coli* [38], and resistance to intestinal colonization by pathogenic *E. coli* compared to pigs receiving milk from non-transgenic goats [23]. Further, a study using 16S rRNA clone libraries and the G2 Phylochip illustrated that hLZ indeed changed the gut microbiome composition, with an increase in the abundance of bacteria associated with gut health (i.e., *Bifidobacteriaceae* and *Lactobacillaceae*) and a decrease in the abundance of those associated with disease (i.e., *Mycobacteriaceae, Streptococcaceae*, and *Campylobacterales*) [22]. In our study, the reduced *E. coli* content in the duodenum implied that rhLZ could efficiently inhibit *E. coli* growth in vivo. However, there was no difference observed in other intestinal segments or in the content of other examined bacteria. We assumed that the rhLZ concentration in the duodenum was much higher than that in other intestinal segments. Regarding rhLZ absorption and degradation, rhLZ concentration decreased to a level that was unable to induce obvious changes. As far as we know, *E. coli* infection is one of the main causes of sucking piglets diarrhea, especially the strain K88 and F18. Since lysozyme can effectively decreased the number of *E. coli* and inhibit *E. coli* infection, the rhLZ transgenic pigs have an important breeding value. In a future study, we plan to analyze the gut microbiome by 16SrRNA sequencing to detect other changes caused by rhLZ-rich milk during different stages of lactation.

The mucosa is one of three major components indicating “gut health” [39] and the morphology of the small intestinal is often used as a marker to estimate intestinal health in pigs [14], [40], [41]. Wider villi in the duodenum and higher villi in the ileum were observed in pigs nursed with goat hLZ milk compared to those reared on control milk, but there were no significant changes in crypt depth in the ileum or jejunum [23], [42]. Similarly, pigs consuming lysozyme (100 mg/kg diet) showed no differences in villus heights or crypt depths in the ileum. However, villus height was increased and crypt depth was decreased in the jejunum, resulting in an increased villus height to crypt depth ratio [14]. The intestinal villus height to crypt depth ratio can significantly affect nutrient digestion and gastrointestinal absorption [39]. A greater villus height to crypt depth ratio corresponds to an increased surface area and is therefore advantageous to nutrient absorption [43]. In our study, a greater villus height to crypt depth ratio was observed among different intestinal segments of piglets nursed by transgenic sows. Furthermore, the changes in intestinal morphology indicated that high hLZ concentrations in milk from transgenic pigs may lead to a greater absorptive capacity and improve small intestinal morphology in sucking piglets. On the other hand, the young piglets is subjected to myriad of stressors with marked changes to the histology of the small intestine, such as villous atrophy and crypt hyperplasia at weaning. Those histology changes cause decreased digestive and absorptive capacity and contribute to post-weaning diarrhea. The improved intestinal morphology of piglets due to rhLZ milk feeding can improved the absorption capacity after weaning and thus may relieve post-weaning diarrhea.

In conclusion, we successfully produced transgenic cloned pigs that produced high concentrations of bioactive rhLZ in milk. High-level rhLZ expression did not change the composition of milk derived from transgenic pigs. The results of the feeding experiments demonstrated that rhLZ-enhanced milk can positively influence intestinal morphology and inhibit the growth of *E. coli* in the duodenum without adversely affecting weight gain or piglet growth. Together, our results laid a good foundation for further studies on the diarrhea-resistant effects of transgenic pigs.

## Supporting Information

Files S1 **Tables S1–S4.**
(DOCX)
