# Supplementary material for: High-Level Recombinant Human Lysozyme Expressed in Milk of Transgenic Pigs Can Inhibit the Growth of Escherichia coli in the Duodenum and Influence Intestinal Morphology of Sucking Pigs
Source: PLoS One. 2014 Feb 21;9(2):e89130. doi: 10.1371/journal.pone.0089130 (PMC3931683; doi:10.1371/journal.pone.0089130)
Supplement: Files S1 — Tables S1–S4. (DOCX) [file pone.0089130.s001.docx]

**Supporting information**

**Table S1. Primers for transgenic pigs verification.**

| Primer name | Primer sequence | Utility |
| --- | --- | --- |
| P-hLZ-395F | 5’-CTCAAATGATCTGCCCACCT -3’ | PCR detection of transgenic founders (395 bp) |
| P-hLZ-395R | 5’-GAAGGGATGAAATCACGGAA-3’ |  |
| P-hLZ-637F | 5’-TTATACACACGGCTTTAC-3’ | PCR digoxigenin-labeled probe synthesis (637 bp) |
| P-hLZ-637-R | 5’-CAGCATCAGCGATGTTATCT-3’ |  |
| P-hLZ-322F | 5’-ATCAGCCTAGCAAACTGGAT-3’ | RT-PCR for hLZ (322 bp) |
| P-hLZ-322F | 5’-CTCCACAACCTTGAACATAC-3’ |  |
| P-GAPDH-421F | 5’-ACCCAGAAGACTGTGGATGG-3’ | RT-PCR for GAPDH control (421 bp) |
| P-GAPDH-421R | 5’-CCCTGTTGCTGTAGCCAAAT-3’ |  |
| P-hLZ-112F | 5’-TGCTGGGTGCCTGAGATTCA-3’ | Q-PCR for hLZ copy number (112 bp) |
| P-hLZ-112R | 5’-AGTTCAAAATGGGAAATAACTGG-3’ |  |
| P-MSTN-110F | 5’-TCTGAGACCCGTCAAGACTCCTA-3’ | Q-PCR for MSTN control (110 bp) |
| P-MSTN-110R | 5’-TGTCAAGTTTCAGAGATCGGATTC-3’ |  |

**Table S2. Expression level and enzymatic activity of rhLZ in the milk of transgenic pigs.**

| **Time** | **Expression of rHLZ(mg/L)** | **Antibacterial Activity (U/mL)** | **Antibacterial Activity(U/mg)** |
| --- | --- | --- | --- |
| **0h** | 140.68±11.92 | -- | -- |
| **6h** | 142.07±26.42 | 107760±32076 | 761330.20±204989.42 |
| **12h** | 121.29±24.89 | 105420±32064 | 866135.85±221285.51 |
| **24h** | 85.01±17.52 | 66132±24009 | 767131.40±213270.39 |
| **48h** | 112.63±10.95 | 82116±13150 | 734039.89±124475.65 |
| **72h** | 122.13±19.89 | 97800±25310 | 792924.63±146169.28 |
| **7d** | 96.05±10.72 | 87576±10173 | 917753.11±125383.06 |
| **14d** | 127.09±15.05 | 110076±28238 | 867732.75±206607.54 |
| **21d** | 100.14±9.34 | 81300±18516 | 808632.66±153703.61 |
| **Mean Value** | 116.34±24.46 | 922723±26413 | 814460.05 ±172647.98 |

Values are averages ±standard deviations. n = 5 gilts at each time.

**Table S3. Enzymatic activity of rhLZ in the milk of non-transgenic pigs.**

| Time | Antibacterial Activity(U/mL) |
| --- | --- |
| 6h | 84.65 ± 6.66 |
| 12h | 69.59 ± 11.40 |
| 24h | 3.65 ± 0.42 |
| 48h | ND |
| 72h | ND |
| 7d | ND |
| 14d | ND |
| 21d | ND |

Values are averages ±standard deviations. n = 4 gilts at each time.

**Table S4. 16 kinds of amino acids in milk of first-parity control and transgenic gilts.**

| Amino Acids | Transgenic | | Non-transgenic | |
| --- | --- | --- | --- | --- |
|  | Colostrum | Milk | Colostrum | Milk |
| Aspartic Acid | 1.14±0.25 | 0.40±0.03 | 1.32±0.17 | 0.39±0.02 |
| Threonine | 0.99±0.20 | 0.20 ±0.02 | 0.99±0.11 | 0.19±0.01 |
| Serine | 1.13±0.24 | 0.25±0.02 | 1.07±0.18 | 0.25±0.01 |
| Glutamic Acid | 2.71±0.40 | 0.95±0.06 | 2.5±0.35 | 0.95±0.07 |
| Glycine | 0.60±0.12 | 0.15±0.01 | 0.58±0.08 | 0.15±0.00 |
| Alanine | 0.75±0.13 | 0.18±0.01 | 0.70±0.09 | 0.17±0.00 |
| Valine | 1.02±0.16 | 0.22±0.01 | 0.96±0.11 | 0.22±0.01 |
| Methionine | 0.25±0.04 | 0.09±0.01 | 0.23±0.02 | 0.09±0.01 |
| Isoleucine | 0.52±0.07 | 0.17±0.02 | 0.48±0.06 | 0.16±0.02 |
| Leucine | 1.61±0.24 | 0.42±0.03 | 1.50±0.23 | 0.42±0.02 |
| Tyrosine | 0.86±0.15 | 0.21±0.02 | 0.81±0.13 | 0.20±0.02 |
| Phenylalanine | 0.78±0.13 | 0.20±0.02 | 0.73±0.11 | 0.20±0.01 |
| Lysine | 1.16±0.19 | 0.34±0.02 | 1.07±0.15 | 0.34±0.03 |
| Histidine | 0.42±0.07 | 0.12±0.01 | 0.39±0.05 | 0.12±0.01 |
| Arginine | 0.85±0.16 | 0.22±0.01 | 0.78±0.11 | 0.22±0.01 |
| Proline | 1.42±0.24 | 0.50±0.03 | 1.32±0.18 | 0.49±0.03 |

No significant differences were detected between transgenic group and non transgenic group (*p*>0.05). Values are averages ±standard deviations. Transgenic, n=6; non-transgenic, n=3.
